# Plasmatic level of neurosin predicts outcome of mild cognitive impairment

**DOI:** 10.1186/1755-7682-1-11

**Published:** 2008-07-11

**Authors:** Manuel Menendez-Gonzalez, Patricia Castro-Santos, Maria Teresa Calatayud, Pablo Perez-Piñera, Renee Ribacoba, Marta Martinez-Rivera, Carmen Gutierrez, Alfonso Lopez-Muñiz, Ana Suarez

**Affiliations:** 1 Internal Medicine Department. Hospital Álvarez-Buylla, Mieres, Spain; 2 Department of Immunology. Hospital Universitario Central de Asturias, Oviedo, Spain; 3 Department of Neurology. Hospital Universitario Central de Asturias, Oviedo, Spain; 4 Department of Functional Biology. Universidad de Oviedo, Oviedo, Spain; 5 Department of Morphology and Cellular Biology. Universidad de Oviedo, Oviedo, Spain

## Abstract

**Background:**

Mild Cognitive Impairment (MCI) is a disorder considered to be a transitional stage from health to dementia. Diagnosis of dementias at these early stages is always troublesome because the pathophysiologic events leading to dementia precede clinical symptoms. Thus, the development of biomarkers that can be used to support the diagnosis of dementias at early stages is rapidly becoming a high priority. We have recently reported the value of measuring plasmatic levels of neurosin in the diagnosis of Alzheimer's disease (AD). The aim of this study is to determine whether measuring plasmatic concentration of neurosin is a valuable test to predict progression of MCI.

**Methods:**

Plasmatic neurosin concentrations were measured in 68 MCI patients and 70 controls subjects. Blood samples were obtained at the beginning of the study. Sixty six patients diagnosed with MCI were observed for 18 months. In 36 patients a second blood sample was obtained at the endpoint.

**Results:**

The mean value of plasmatic neurosin concentration differs significantly between MCI patients who converted to Dementia with vascular component, those who converted to AD, or those who remained at MCI stage. The relative risk of developing Dementia with vascular component when neurosin levels are higher than 5.25 ng/ml is 13 while the relative risk of developing mild AD when neurosin levels are lower than 5.25 ng/ml is 2. Increases in the levels of neurosin indicate progression to Dementia with vascular component.

**Conclusion:**

The measurement of plasmatic neurosin level in patients diagnosed with MCI may predict conversion from MCI to Dementia with vascular component. A single measurement is also valuable to estimate the risk of developing AD and Dementia with vascular component. Finally, repeated measurement of plasmatic neurosin might be a useful test to predict outcome in patients with MCI.

## Background

Worldwide, the prevalence of dementia exceeds 24 million people and, more importantly, current trends predict that the number of people with dementia will double every 20 years [1] increasing not only monetary cost in medical terms, but also social awareness and concern about these diseases. Currently, there are no laboratory tests that allow distinguishing reliably different types of neurodegenerative dementias and, therefore, their diagnosis remains based on clinical criteria once other "treatable causes" of dementia have been discarded. For these reasons, increasing effort is invested in developing biomarkers useful to precisely diagnose different types of dementia, to allow diagnosis at earlier stages, and to monitor progression of the dementia and response to treatment. In this regard, peripheral fluids as source for biological markers may be of great importance because of their easy accessibility.

The pathophysiologic events leading to neurodegeneration begin long before clinical symptoms arise but diagnosis at early stages is always troublesome. Patients with mild cognitive impairment (MCI), a condition considered a transitional stage from health to dementia, have increased risk of developing AD [2] although they may, in fact, develop any neurodegenerative disease or stay at the same stage without further cognitive deterioration. The identification of biomarkers to predict the onset of AD or other dementias in presymptomatic patients is highly important in critical practice because these diagnostic tests could be extremely useful to improve diagnostic accuracy and monitor the efficacy of putative therapies.

Human kallikreins are a multigene family of 15 secreted serine-type proteases aligned in tandem in chromosome 19q13.4. Neurosin (Kallikrein 6) is a secreted "trypsin-like" serine-protease mainly expressed in the brain [3-5]. Levels of neurosin in brain tissue, CSF, and blood are altered in AD thus suggesting that measurement of neurosin levels could potentially contribute to better diagnose dementias. Some of neurosin's substrates include myelin basic protein, plasminogen, α1-antitrypsin, and amyloid precursor protein (APP) [6,7]. Interestingly, neurosin has been shown to cleave APP to generate amyloid beta peptides [8].

In a previous study we measured plasmatic levels of neurosin in healthy individuals and patients with cognitive symptoms independently of what the final diagnosis was. We found that plasmatic neurosin concentration increases with age in healthy people and decreases in patients with AD. We also showed that plasmatic neurosin concentration is lower in patients with AD than in patients with Vascular Dementia [9].

The aim of the studies described in the current manuscript was to determine whether measuring plasmatic concentration of neurosin in MCI patients is a valuable test to predict conversion to dementia.

## Methods

This project has been approved by the Research and Ethics Committees of the Hospital Universitario Central de Asturias (HUCA). Informed consent was obtained from all individuals or their guardians. We have followed the recommendations from the Standards for Reporting of Diagnostic Accuracy Group (STARD initiative) [10].

### Patients

#### Screening

We screened patients assessed at the HUCA-Dementia Unit who had symptoms suggestive of MCI.

#### Inclusion criteria

Patients suffering MCI according to Petersen's criteria [11].

#### Exclusion criteria

Patients suffering from any kind of vascular or degenerative dementia. Patients suffering from "treatable cognitive impairment" including cancer (abnormal levels of kallikreins), hydrocephalus, deficiency or toxic disease, or attributable to any other disorder. Renal or hepatic dysfunction (often associated with abnormal proteins levels).

#### Controls

Individuals without any central nervous system disorder and ageing more than 60 years old were included as controls. Controls were recruited among healthy people matching the age of patients, mainly the spouses of the patients included, or patients studied in our department for peripheral nervous system diseases.

#### Clinical Assessment

All patients were studied with neuroimaging and full neuropsychological assessment following the AAN recommendations [12]. Neuropsychological assessment included the "Test Barcelona Abreviado" [13] in all patients as well as other tests depending on each patient's profile. A functional scale (The Barthel Index) [14] was also performed.

#### Follow-up

Patients were observed for 18 months with visits every 6 including functional assessment. Neuroimaging and full neuropsychological assessment were performed again at the endpoint and the diagnosis was reassessed.

#### ELISA to detect neurosin

The level of neurosin was determined at baseline in controls and patients and at the endpoint in 36 patients using ELISA (human kallikrein 6 research ELISA, IBEX, Canada) following manufacturer's instructions. This test is based on an assay previously described by Diamandis et al. [15]. Briefly, we performed a two step sandwich immunoassay using recombinant neurosin as standard, a capture mouse anti-neurosin monoclonal antibody and a mouse anti-neurosin biotin-conjugated monoclonal antibody, both within a stabilizing matrix including BSA. Bound biotinylated antibody was detected with streptavidin-horseradish peroxidase conjugate and tetramethylbenzidine as substrate. The two anti-neurosin mouse monoclonal antibodies used in this assay are specific for neurosin and showed no cross reactivity against purified recombinant human kallikrein 2, 3 (PSA), 5, 8, 10 and 13. Intra-assay precision of this ELISA is CV ≤ 3.97 and inter-assay precision is CV ≤ 7.22. Standard curves were generated with recombinant neurosin standard dilutions between 0.2 and 20 ng/ml.

### Statistics

A Chi-square test was performed to study gender distribution differences between controls and patients. A T-Student test was performed to assess differences in age between controls and patients. A T-Student was performed to assess differences between neurosin in patients diagnosed with MCI and controls in the initial group. ANOVA test and Bonferroni correction were performed to asses the differences between the three groups after follow up. A T-Student test was performed to assess the differences generated with time within every final diagnostic group. Chi-square tests were performed to determine whether there was an association between the occurrence of clinical progression and the dichotomised plasmatic levels of neurosin. Logaristic regression methods controlling gender and age were performed to determine the probability of developing dementia in function of the dichotomised plasmatic levels of neurosin.

## Results

### Sample description

We initially included in the study 70 control individuals (29 males and 41 females) and 68 patients with MCI (21 males and 47 females). Eleven patients were screened but excluded after assessment since they met one or more of the exclusion criteria.

All patients and controls were Caucasian older than 60 years. To evaluate whether age or gender could be variables leading to misinterpretation of results, we analyzed the composition of the two study groups. The results demonstrated that there were no significant differences in gender distribution (p = 0.760) nor in mean age (p = 0.454).

### Diagnostic evolution of MCI patients

Sixty six patients with MCI were followed up 18 months after diagnosis. The MCI did not progress in 30 cases (45.4%). Twenty three patients (34.8%) developed AD (according to the NINCDS-ADRDA criteria [16]) and thirteen patients (19.76%) developed Dementia with vascular component (probable or possible vascular dementia according the NINCDS-AIREN criteria [17]). Therefore, the conversion rate was 36.4% is per year.

### Plasmatic concentration of neurosin in MCI patients and Controls

**Figure 1:**
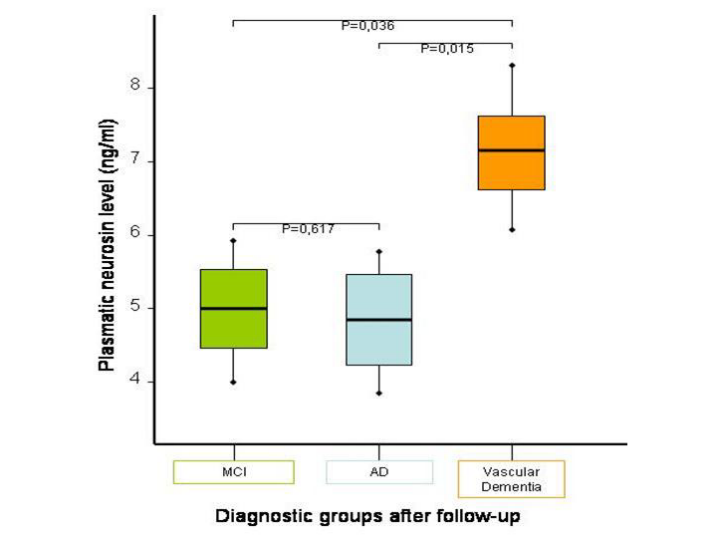
**Differences in plasmatic level of neurosin between the three diagnostic groups after follow up**. The plasmatic neurosin concentration is shown for the three final diagnostic groups (MCI in green, AD in blue and Dementia with vascular component in brown). Statistical differences were found between Dementia with vascular component and both AD and MCI.

The mean value of plasmatic neurosin was not significantly different (p = 0,348) between controls (mean: 5.52 ng/ml) and MCI patients at the beginning of the study (mean: 5.97 ng/ml) (Table 1). However, the analysis of the group of 66 patients that were followed up for 18 months shows that when we retrospectively calculated the mean values of plasmatic concentration of neurosin in MCI patients who converted to each group there were significant differences between those who converted to Dementia with vascular component (mean: 7.17 ng/ml), those who converted to AD (mean: 4.96 ng/ml) (p = 0.015), and those who remained at MCI stage (mean: 5.03 ng/ml) (p = 0.036) (Figure 1). There were no differences between those who did not convert and those who converted to AD (p = 0.617).

**Table 1:** Plasmatic neurosin concentration in the final diagnostic groups.

*Final Diagnosis*	*Initial value (ng/ml)*			*Final value (ng/ml)*		
	
	*N*	*Mean*		*N*	*mean*	*95% confidence interval*
*MCI*	30	5.03	(4.06–6.00)	6	5,56	(4.69–6.43)
*AD*	23	4.96	(4.08–5.84)	19	4,87	(4.42–5.32)
*Dementia Vasc. comp.*	13	7.17	(6.12–8.22)	11	8,79	(8.28–9.30)

Initial and final data of neurosin concentration for the three final diagnostic groups.

### Evolution of plasmatic levels of neurosin predict outcome in patients with MCI

**Figure 2:**
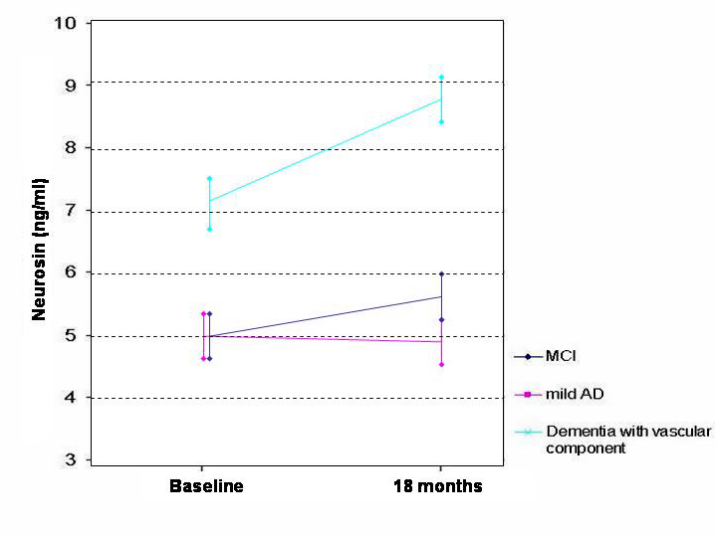
**Evolution with time of the plasmatic level of neurosin**. Colour legend shows diagnostic groups after follow up. Vertical bars show standard deviation at baseline and final (after 18 months) points.

To determine whether time-dependent variation of plasmatic levels of neurosin in patients with MCI is predictive of outcome, we also measured plasmatic concentrations of neurosin in 36 patients at the endpoint of the study (Table 1 and Figure 2). The results showed that in only 18 months the levels of neurosin significantly increased (from 7.17 ng/ml to 8.79 ng/ml) in patients whose disease progressed to dementia with vascular component (p = 0.043) while the decrease in patients whose disease progressed to AD (from 4.96 ng/ml to 4.87 ng/ml) was not statistically significant (p = 0.747).

### Plasmatic levels of neurosin and risk of conversion

To further analyze these findings, we classified patients according to their initial neurosin concentration in low (≤ 5.25 ng/ml) or high (>5.25 ng/ml) (Table 2) and determined whether there is an association between disease evolution and plasmatic levels of neurosin. We set the cut-off at 5.25 ng/ml because it is the best cut point previously determined by us to diagnose AD [9]. Results demonstrated that, in patients diagnosed with MCI, there is an association between the variable "developing Dementia with vascular component" and a plasmatic concentration of neurosin higher than 5.25 ng/ml (p < 0.001) as well as an association between the variable "developing AD" and a plasmatic neurosin concentration lower than 5.25 ng/ml (p = 0.004). There is no association between "no conversion" and dichotomised levels of neurosin (p = 0.437).

**Table 2:** Final diagnostic distribution after the 18 months follow up period.

	>5.25	≤ 5.25	P (χ^2 ^test)
MCI	12 (40%)	18 (60%)	0.437
AD	7 (30%)	16 (70%)	0.004
DVasc.Comp	12 (93%)	1 (7%)	<0.001

Patients initially diagnosed with MCI were followed up for 18 months. Data show final diagnostic distribution in relation to their initial plasmatic levels of neurosin (≤ or > 5.25 ng/ml) and the association (p) value.

Then we calculated the probability of conversion. The risk of developing Dementia with vascular component for MCI patients with plasmatic neurosin higher than 5.25 ng/ml is 13.10 (with an interval 11.92–14.32 for a confidence of 95%). The relative risk of developing AD in patients with MCI and neurosin levels lower than 5.25 ng/ml is 2.10 (with an interval 1.32 – 2.92 for a confidence of 95%).

## Discussion

It is now accepted that neurosin concentration in brain tissue of patients with AD is decreased compared to normal individuals [18,19]. Whether the levels of neurosin in CSF and plasma increase [20] or decrease [21,22] was an active controversy. To address those discrepancies, we recently studied plasmatic concentration of neurosin in different types of dementia compared with normal individuals. We demonstrated that plasmatic levels of neurosin increase in normal individuals as they get older [9]. More importantly, in that study we also demonstrated that changes in plasmatic concentration of neurosin may be valuable to predict outcome in certain types of dementias.

We studied here whether measuring plasmatic concentration of neurosin may be a valuable test to predict progression of MCI. We measured plasmatic levels of neurosin in controls and patients diagnosed with MCI. MCI patients were followed for 18 months and then plasmatic concentrations of neurosin were measured again at the endpoint in 36 patients (Table 1).

Our results, although preliminary, may be potentially very significant in the prognosis of MCI for the following reasons: 1) the mean value of plasmatic neurosin did not significantly differ between MCI and controls but it differs between patients with MCI who converted to Dementia with vascular component and those who converted to AD or remained at MCI stage. 2) Repeated measurement of plasmatic neurosin is potentially a good parameter to predict progression in patients with MCI since there is a time-dependent association between plasmatic levels of neurosin and prognosis: increasing levels of neurosin are predictive of conversion to Dementia with vascular component. 3) The risk of conversion is associated with levels of neurosin: the relative risk of developing Dementia with vascular component when neurosin level is lower than 5.25 ng/ml is close to zero; however, the relative risk of developing mild AD when neurosin levels are lower than 5.25 ng/ml is 2. On the other hand, the relative risk of developing Dementia with vascular component when neurosin level is higher than 5.25 ng/ml is above 13.

The mechanism by which plasmatic concentration of neurosin increases in patients that convert to Dementia with vascular component remains unknown. One possible explanation is the interaction of neurosin with PARs (proteinase-activated receptors). PARs comprise a family of G protein-coupled receptors activated by thrombin or other coagulation or inflammatory proteases released at sites of tissue injury [23]. PARs are widely expressed in the CNS; similar to hK6, the highest densities of PAR 2 were observed in the hippocampus, cortex, amygdala, thalamus, hypothalamus, and striatum [24]. PAR 2 is activated by neurosin [25,26]. Among other actions, PAR-2 induces arterial and venous dilatation *in vivo *and therefore could contribute to blood pressure dysregulation, which, in turn, may account for the development of Dementia with vascular component [27,28].

In conclusion, we report for the first time that measuring plasmatic neurosin concentration may be useful to predict conversion of patients diagnosed with MCI and we established the relative risks of developing AD and Dementia with vascular component according with the plasmatic level of neurosin.

## Competing interests

The authors declare that they have no competing interests.

## Authors' contributions

MMG and PCS contributed to conception and design, analyzed the data and drafted the manuscript. MMG, MM and MC made the clinical assessment. ACS, AS and CG made the laboratory work. PPP and RR contributed to data analysis and critically reviewed the manuscript for intellectual content. MMG, CG, AS and ALM designed and managed the overall study, interpretation of the results and critically reviewed the manuscript for intellectual content. All authors read and approved the final manuscript.
